# The Organ-Joint Axes in Osteoarthritis: Significant Pathogenesis and Therapeutic Targets

**DOI:** 10.14336/AD.2024.1223

**Published:** 2025-11-21

**Authors:** Dinglong Yang, Yujing Chen, Junfei Guo, Xin Xu, Mingyi Yang, Jiale Xie, Ke Xu, Peng Xu

**Affiliations:** ^1^Department of Joint Surgery, Honghui Hospital, Xi’an Jiaotong University, Xi’an, 710054, China.; ^2^Xi'an Key Laboratory of Pathogenesis and Precision Treatment of Arthritis, Xi’an, 710054, China.; ^3^Xijing 986 Hospital, Fourth Military Medical University, Xi'an, 710054, China.

**Keywords:** Osteoarthritis, brain, gut microbiota, sarcopenia, skeletal interoception

## Abstract

Osteoarthritis (OA), a prevalent age-related disease, is increasingly recognized as a multifactorial condition. This comprehensive review provides a multifaceted perspective on the organ-joint crosstalk contributing to OA, transcending the traditional focus on local joint pathology. Based on current research, we discussed the brain-joint, gut-joint, muscle-joint interactions in the etiology and progression of OA. In brain-joint axis, the neuroendocrine regulation, circadian rhythms, and leptin signaling influence joint tissues. We also discussed the role of prostaglandin E2 in skeletal interoception and its potential as a therapeutic target. The gut-joint axis is underscored by the impact of gut microbiota dysbiosis on systemic inflammation and metabolic disorders, both of which are implicated in OA pathogenesis. Furthermore, age-related sarcopenia, characterized by muscle mass and strength loss, is identified as a significant risk factor. Sarcopenia may contribute to OA progression through compromised mechanical support, systemic inflammation, and muscle-derived myokines. Finally, we synthesize the evidence supporting the modulation of circadian rhythm, skeletal interoception, gut microbiome, and muscle mass as innovative strategies for OA management. The organ-joint crosstalk is integral to the complex pathogenesis of OA, highlighting the multifactorial nature of OA and the potential for targeted therapeutic interventions. By integrating these multidimensional perspectives, we aim to enhance our understanding of OA pathogenesis and explore potential pharmacological targets.

## 1. Introduction

The skeletal system stands as a metabolic organ, serving as the structural scaffold that underpins the entire physique [1]. Beyond its well-documented role in hematopoiesis, it operates as a pivotal endocrine entity, orchestrating the intricate balance of glucose, lipids, and minerals throughout the body [2, 3]. Engaging in a sophisticated metabolic dialogue with other organs, the skeleton is not merely a passive participant but actively modulates the metabolic processes of other tissues, exemplified by its influence on hepatic cholesterol metabolism [4]. Conversely, hepatic and muscular tissues, among others, release factors that reciprocally maintain the equilibrium of bone homeostasis [5, 6]. The human body, an integrated and complex organism, is critically dependent on the intricate interactions among its organs to sustain homeostatic balance. The burgeoning field of organ-organ axis research is gaining prominence among the scientific community, providing a more robust and holistic approach to understanding the etiology of diseases, transcending the limitations of localized molecular biological studies.

Osteoarthritis (OA) is one of the most prevalent orthopedic diseases affecting over 300 million individuals globally [7, 8]. In China, the prevalence of OA is striking among the elderly population; 62.2% of individuals over 60 are affected, this figure escalates to 80% for those aged 75 and older, with over 8% of the total population enduring symptomatic OA [9]. OA's pathophysiology, a principal contributor to physical disability, remains largely enigmatic [10]. OA is hallmarked by the degeneration of cartilage, implicating subchondral bone, ligaments, menisci, synovium, and joint capsules, all of which are accompanied by persistent arthralgia [11]. OA has a multifactorial etiology that encompasses inflammation, metabolic dysregulation, biomechanical stress, and genetic predispositions. This complicates the efficacy of conservative therapeutic interventions. At present, joint replacement surgery represents the most efficacious treatment for advanced OA [12]. Historically, research has predominantly targeted the local pathogenic mechanisms within the cartilage, subchondral bone, and synovium of OA, yet our comprehension of the disease underpinnings remains constrained [13, 14]. In the human body, joint tissues are subject to the regulation of other organs. Consequently, it is crucial to delve into the systemic regulatory dynamics involving OA, rather than confining research to the localized pathogenic mechanisms. This approach is essential for a more comprehensive understanding of OA multifaceted etiology and for the development of more effective therapeutic strategies.

In recent years, research has uncovered significant interactions between the brain, gut, muscles, liver, lungs, and other organs with the skeletal system, which have a profound impact on bone metabolism and associated pathologies [5, 6, 15-18]. Joint tissues, a vital part of the skeletal system, are also subject to the influence of other internal organs in homeostasis and disease progression [19, 20]. Notably, more and more researches highlight the role of subchondral bone alterations in the advancement of OA, with evidence suggesting that these alterations may occur even before damage to the articular cartilage [21]. The study of organ interactions has broadened our understanding of OA, presenting a more holistic view of this disease. It will open up extensive and promising avenues for elucidating the pathogenesis of OA and the discovery of novel therapeutic approaches.

In this review, we initially provide an overview of the pathological changes in OA, including cartilage degeneration, aberrant subchondral bone remodeling, and synovial inflammation. This serves to establish a foundational understanding for the reader regarding the subsequent influence of organ-joint crosstalk on OA pathophysiology. Subsequently, we delve into the role of organ-joint interactions in the etiology of OA. Based on current evidence, we emphasize the brain-joint, gut-joint, and muscle-joint axes in OA. Concluding our discourse, we investigate the potential therapeutic efficacy of interventions that target these organ-joint interactions. This comprehensive review delves into the etiology of OA through a broader lens, highlighting the pivotal role of multi-organ interactions in OA progression and their implications for therapeutic strategies.

## 2. The pathological changes in OA

Traditionally, the primary pathological focus in OA was the degeneration of articular cartilage. The contemporary view, encompasses a more holistic perspective, acknowledging OA as a disease that implicates the entire joint structure, including cartilage, synovium, subchondral bone, ligaments, and menisci [22]. In this section, we aim to provide an overview of the pathological alterations occurring in the cartilage, subchondral bone, and synovium as OA progresses, thereby laying the groundwork for a deeper understanding of the subsequent discussions in this review.

## 2.1 Cartilage degeneration

Articular cartilage is an elastic tissue that lines the joint surfaces, composed mainly of chondrocytes and extracellular matrix (ECM). The ECM is complex, predominantly consisting of collagen and proteoglycans [23]. This tissue is endowed with resilience, enabling it to withstand and dissipate mechanical loads from the joint [24]. However, factors such as senescence, aberrant mechanical loading, inflammatory processes, and oxidative stress can disrupt the metabolic balance of chondrocytes, resulting in catabolism over anabolism. This shift leads to the progressive degradation of the cartilage matrix, finally exposing the subchondral bone [25-28]. The lack of blood vessels and nerves in articular cartilage markedly hampers the capacity for self-repair, relying instead on synovial fluid and adjacent tissues for nutrition. This inherent limitation narrows the therapeutic options for the treatment of cartilage degeneration [29]. Moreover, the deterioration of cartilage intensifies joint inflammation, which furthers tissue damage. Synovial fluid, which originates from the synovial membrane, reflects the metabolic state of the body to some extent and acts as a medium for the interaction between internal organs and articular cartilage [30]. Deep to the cartilage lies the calcified cartilage, demarcated from the hyaline cartilage by the tidemark. This calcified layer acts as a critical interface between cartilage and subchondral bone, a diminished ratio of hyaline to calcified cartilage thickness is indicative of the advancement of OA [31].

## 2.2 Abnormal subchondral bone remodeling

The subchondral bone encompasses the bone plate and trabecular bone located beneath the calcified cartilage [32]. The subchondral trabeculae are porous structures that are richly vascularized and innervated. Beyond their role in cushioning mechanical loads and providing structural integrity, these structures also serve as vital suppliers of nutrition for the cartilage [33, 34]. In the subchondral bone microenvironment, bone marrow mesenchymal stem cells (BMSCs) and their differentiated osteoblasts (OBs), monocytes/macrophages (Mo/Macs) and their differentiated osteoclasts (OCs), and osteocytes are the main cellular constituents. They not only interact with each other but also interact with chondrocytes through micro-fractures and other pathways to maintain joint homeostasis [12, 35-37]. When subjected to detrimental influences like abnormal mechanical stress, the subchondral bone manifests pathological alterations associated with OA. Throughout the disease progression, both osteogenic and osteoclastic activities in the subchondral bone are elevated. In the early stage of OA, bone resorption exceeds bone formation, leading to thinning of the bone plate and increased separation of the trabeculae. In the late stage of OA, bone formation activity gradually exceeds resorption activity, resulting in thickening of the bone plate and trabeculae, and the formation of osteophytes [21, 38]. Although bone mass in the subchondral bone increases in late-stage OA, the degree of mineralization and elasticity is reduced. This abnormal remodeling of the subchondral bone leads to a weakened ability to absorb loads, thereby increasing the abnormal mechanical loads on the cartilage and causing degeneration [39]. It is also observed that the augmented osteoblastic and osteoclastic activities in the subchondral bone are often correlated with an increased distribution of calcitonin gene-related peptide (CGRP)+ sensory nerves and type H vessels. The promoted innervation of CGRP+ sensory nerves partially elucidates the mechanisms underlying the OA pain [38, 40]. While many studies have shed light on the interaction mechanisms between internal organs and bone tissue, the subchondral bone emerges as a potential nexus for interactions between OA and internal organs.

## 2.3 Synovial inflammation

The synovium is a thin layer of tissue lining the innermost surface of the joint cavity. It plays a role in lubrication, nutrition, and removal of metabolic byproducts within joints by secreting synovial fluid. The normal synovium, composed of fibroblasts and macrophages, performs specific functions within the joint system, including host, immune modulation, and secretion of lubricating fluids [41, 42]. The inflamed synovial tissue exhibits inflammatory edema, fibrosis, and thickening [43]. A growing body of evidence indicates that in patients with OA, the synovial tissue exhibits inflammation-related characteristics, including neovascularization, infiltration of macrophages and lymphocytes, and synovial hyperplasia [44-46]. Overproduction of pro-inflammatory mediators, such as interleukin (IL)-1β and tumor necrosis factor-α (TNF-α), is observed, originating from pro-inflammatory macrophages and fibroblasts [47]. Synovial inflammation is associated with OA symptoms and progression [48]. Other organs also interact with synovial tissue, thus contributing to the development of synovial inflammation in OA.

## 3. The brain-joint crosstalk in the pathogenesis of OA

## 3.1 The neuroendocrine regulation of OA

In this section, we will discuss the role of substances secreted by the central nervous system (CNS) in the homeostasis of subchondral bone, cartilage metabolism, and synovial inflammation in OA.

The neuroendocrine system represents a sophisticated integration of the nervous and endocrine systems' functions. Within this intricate system, neurons exhibit the dual properties of neural and endocrine cells. Through neurotransmitters and hormonal mediators, the neuroendocrine system plays a pivotal regulatory role in bone metabolism, with particular emphasis on the metabolic activities of subchondral bone. This system contributes to the fine-tuning of subchondral bone equilibrium, involving peptides derived from proopiomelanocortin (POMC), including α-melanocyte-stimulating hormone (α-MSH), adrenocorticotropin (ACTH), and β-endorphin (β-ED), as well as sympathetic neuropeptides such as vasoactive intestinal peptide (VIP) and neuropeptide Y (NPY). OCs, OBs, and osteocytes exhibit a range of neuropeptide receptors, such as melanocortin receptors (MCRs) and receptors for VIP and pituitary adenylate cyclase-activating polypeptide (PACAP) [49]. Type H vessels have been confirmed as an important part in the pathological changes of OA subchondral bone. The CGRP+ cells dilate blood vessels and stimulate the migration of endothelial cells, promoting angiogenesis in bone remodeling [50]. Multiple neuropeptides play a role in this process, such as substance P (SP), NPY, and VIP [51-53]. The autonomic nervous system (ANS) regulates bone homeostatic. Generally, the activation of the sympathetic nerve system (SNS) leads to bone resorption while suppressing bone formation. Conversely, the parasympathetic nervous system (PSNS) exerted opposing effects through acetylcholine. Under normal conditions, a delicate balance is maintained between subchondral bone resorption and formation through the coordinated actions of the SNS and PSNS. Advanced OA is characterized by an imbalance in osteogenic and osteoclastic activities [38]. For instance, the SNS exerts its physiological effects by releasing norepinephrine (NE). Beta2-Adrenoceptor (β2-AR) is one of the receptors of NE. OA mice with deficiency in β2-AR (Adrb2-/-) exhibit increased subchondral bone plate thickness, enhanced OB activation, reduced OC activation, and an elevated body weight and fat content [54]. These findings may be associated with the progression of OA.

The synovial fluid is essential for joint nutrition, lubrication (through the secretion of hyaluronic acid), and the clearance of metabolic waste [55, 56]. SP, as a neuropeptide, is upregulated in the synovial fluid of OA patients and has a catabolic effect on articular cartilage. Transforming growth factor-beta (TGF-β) notably enhances the synthesis of SP in synovial fibroblasts [57]. Acting on the neurokinin-1 receptor (NK1R) present on chondrocytes, SP incites the production of matrix metalloproteinase-13 (MMP-13), while concurrently suppressing the deposition of proteoglycans within human cartilage [58]. However, there is a significant age difference between the donors of OA chondrocytes and normal chondrocytes in this study. The donors of healthy chondrocytes lack typical age-related phenotypes, including senescence and apoptosis, which may potentially impact the results of this study. Overall, in synovial cells, SP is described as an effective mediator of inflammation by promoting the secretion of prostaglandin E2 (PGE2), various matrix metalloproteinases (MMPs), reactive oxygen species (ROS), IL-1, and TNF-α. The increased level of SP within the synovium and synovial fluid not only augments the conduction of pain signals through synovial sensory neurons but also accelerates cartilage degradation by intensifying chondrocytes catabolism [59-61].

**Table 1:** The neuroendocrine regulation in OA.

Names	Roles
**α-MSH**	Inhibit the release of matrix-degrading enzymes and aggrecanases from OA synovial fibroblasts; suppress the expression of MMP-2 and MMP-13 induced by cytokines; promote the production of cartilage collagen II and aggrecan
**ACTH**	Promote the proliferation of chondrogenic cells and facilitate the differentiation of progenitor cells into chondrocytes
**VIP**	Relieve OA by inhibiting NF-κB signaling pathway; suppress the degradation of cartilage ECM
**NPY**	Promote chondrocyte hypertrophy and cartilage degradation; elevated levels in the synovial fluid of OA patients; associated with the Wantanabe pain score in OA
**PACAP**	Enhance the production of collagen II and inhibit collagen IX and X in chondrocytes
**CGRP**	Promote chondrocyte senescence and apoptosis; suppress the expression of chondrogenic markers
**SP**	Mediate OA joint pain; promote synovial inflammation and cartilage degeneration; accelerate chondrocyte apoptosis and senescence; contribute to angiogenesis in the subchondral bone of OA
**NE**	α1-AR: induce chondrocyte apoptosis; α2-AR: inhibit chondrogenic differentiation of synovial adipose stem cells; β-AR: inhibit cartilage catabolism; β2-AR: β2-AR deficiency promotes calcified cartilage thickening and abnormal subchondral bone remodeling

Although articular cartilage is devoid of nerve fibers, chondrocytes possess a multitude of neurotransmitter receptors, rendering them vulnerable to neuroendocrine signals from surrounding joint tissues. Neurotransmitters modulate chondrocyte metabolism and phenotype by engaging with their specific receptors. The role of SP in chondrocytes has garnered considerable attention. It has been observed that 1 μmol/L of SP induces hyperpolarization of the chondrocyte membrane. The use of a chemical antagonist against the NK1R effectively inhibits the response of chondrocytes to mechanical stimuli, indicating that SP may participate in the transmission of mechanical signals in cartilage through the NK1R [62]. However, the methods used in this study are relatively simple. They require the detection of SP and NK1R expression in the medial and relatively normal lateral tibial plateau cartilage specimens from patients undergoing joint replacement surgery. Alternatively, the cyclic mechanical loads could be applied to mouse joints to verify this conclusion in vivo experiments. Current scholarly consensus posits that SP generally influences chondrocytes in a paracrine fashion, fostering catabolic metabolism and the degeneration of cartilage. SP, produced by synovial fibroblasts, can induce the release of inflammatory mediators, suppressing the expression of chondrogenic markers, and accelerating chondrocyte apoptosis and senescence. The potential catabolic effects of SP may arise from the activation of the extracellular regulated protein kinase (ERK) signaling pathway, which could be mitigated by an enhanced cyclic adenosine monophosphate (cAMP) response [57, 58]. In OA progression, new blood vessels breach the subchondral bone-cartilage boundary, with sensory and sympathetic nerve fibers infiltrating the affected cartilage alongside these vessels [63]. Consequently, neurogenic SP may modulate chondrocyte metabolism by interacting with the NK1R on chondrocytes. The abundance of α-CGRP+ sensory nerves in articular tissues has been well-documented. Notably, α-CGRP has been demonstrated to augment apoptosis and senescence in OA chondrocytes, concurrently suppressing the expression of chondrogenic markers [58]. Furthermore, a significant elevation of cAMP and cyclic guanosine monophosphate (cGMP) has been observed within chondrocytes, which can be reversed by CGRP receptor antagonists [64]. NE, a neurotransmitter released by SNS, impacts the inflammatory response and cellular metabolism of chondrocytes in a dose-dependent manner. The SNS appears to have a dualistic role in OA pathophysiology. At a concentration of 10^-6 M, NE acting on β-adrenergic receptor (AR) receptors counteracts the effects induced by IL-1β on IL-8, MMP-13, glycosaminoglycan (GAG), and type II collagen [65]. Hyun Sook Hwang has corroborated these findings and extended the understanding by showing that β-AR activation significantly influences the phosphorylation of c-Jun N-terminal kinase (JNK) and extracellular regulated protein kinase (ERK) in response to IL-1β, thus preserving a stable chondrocyte phenotype [66]. In contrast, at a lower concentration of 10^-8 M, NE induces chondrocyte apoptosis through α1-AR signaling, contributing to the advancement of OA pathology [65] (Table 1).

## 3.2 Circadian rhythm

Circadian rhythm is the intrinsic biological rhythm that refer to the cyclical fluctuations in life activities with a period of 24 hours [67]. The sleep-wake cycle is a quintessential example of circadian rhythm. The circadian clock is situated in the hypothalamic suprachiasmatic nucleus (SCN). It orchestrates rhythmic signaling to different brain areas, impacting feeding behaviors, motor activities, thermoregulation, and hormonal secretion, thus governing the sleep-wake and activity-rest cycles across a 24-hour period [68, 69]. The articular cartilage, synovium, subchondral bone, and tendons are particularly affected by the daily rest-activity cycle, which is the peripheral circadian clock. Transcriptomic analysis of murine sternum chondrocytes under diurnal rhythms has identified 615 genes (3.9% of the total) with circadian expression patterns [70]. Circadian rhythm disruption (CRD) influences the gene expression of chondrocytes and cartilage tissues, which is confirmed both in vitro and in vivo. Song et al. established a chronic CRD model in 8-week-old Sprague-Dawley (SD) rats by alternating light/dark cycles every 12 hours for 22 weeks. They observed that chronic CRD disrupted the normal anabolic and catabolic balance of cartilage tissue and chondrocytes, promoting cartilage degeneration, which is associated with the activation of the canonical Wnt/β-catenin signaling pathway [71]. Mechanistically, CRD activated the expression of the chondrocyte clock gene *Period 1 (Per1)*, thereby promoting the progression of temporomandibular joint arthritis through the GSK3β/β-catenin pathway [72]. In OA patients, the expression of clock genes in chondrocytes is altered, including increased *Per2* gene expression and decreased *brain and muscle ARNT-like protein-1* (*Bmal1)* gene expression. Knockdown of *Bmal1* exhibited high expression of chondrocyte MMP13 [73, 74]. Similarly to *Bmal1*, mutations in *circadian locomotor output cycles kaput (Clock)* also promoted cartilage degradation and the expression of pro-inflammatory mediators by mediating post-transcriptional regulation of nuclear factor kappa-light-chain-enhancer of activated B cells (NF-κB) [75]. The research by Michal et al. has demonstrated that the circadian clock in femoral head cartilage and intervertebral disc tissues can be reset by periodic mechanical stress, possibly mediated by hypertonic conditions during mechanical loading [76]. This study is indeed intriguing. However, it is important to note that there is currently a lack of direct measurement of the precise mechanical load environment within murine articular cartilage, which may necessitate the future development of an in vivo direct mechanical loading system to further refine this research. The synchronization of daily joint loading and unloading with local gene expression and physiological alterations is likely a crucial aspect of maintaining joint homeostasis. However, peripheral circadian clocks are subject to indirect modulation by the central SCN via neuronal pathways, hormonal secretions, body temperature, and physical activity. The diurnal fluctuations in serum levels of hormones regulated by the brain, such as parathyroid hormone (PTH), have been observed [77]. Adrien and colleagues have discovered that the treatment with PTH prior to cyclic joint loading in mice can significantly improve cartilage health and mitigate the development of load-induced OA [78]. This finding underscores the indirect regulatory influence of the brain on peripheral circadian rhythms. The SCN may also transmit regulatory signals to local cartilage, bone, or ligament tissues through SNS, and the modulation of glucocorticoid and leptin levels [79]. Considering the significant impact of subchondral bone changes on the advancement of OA, circadian clocks are hypothesized to influence the disease trajectory by modulating the homeostasis of subchondral bone. Francis et al. have authored an extensive review encapsulating the influence of brain-regulated systemic rhythms on OA. Owing to space constraints within this summary, readers are directed to the comprehensive review for an in-depth exploration of the topic [80].

## 3.3 Leptin: a potential mediator of crosstalk between joint and brain

Leptin is a polypeptide hormone secreted by adipose tissues. Leptin exerts its effects by binding to leptin receptors (LepR) within the CNS, thereby regulating behaviors and metabolic processes [81, 82]. Leptin levels in the subchondral bone, cartilage, synovial fluid are greater in OA patients than that in healthy people, and this adipokine exerts a proinflammatory effect in synovial fibroblasts [83-85]. In pathological conditions, OBs in OA subchondral bone produce leptin [86]. Previous studies have found that leptin regulates bone homeostasis through neuronal and circulatory pathways in the hypothalamus, which may be involved in subchondral bone remodeling. Leptin binds to ObRb receptors in the brainstem, activating serotonergic neurons to release serotonin. Subsequently, serotonin binds to the 5-hydroxytryptamine (5-HT)2C receptors on ventromedial nucleus of the hypothalamus (VMH) neurons, leading to alterations in bone mass. Furthermore, leptin enhances the expression of cocaine- and amphetamine-regulated transcript (CART) in the arcuate nucleus (ARC) of the hypothalamus, which subsequently inhibits bone resorption. Neuromedin U (NMU) acts as a downstream mediator of leptin-mediated modulation of bone mass at the paraventricular nucleus (PVN) [81]. These findings indicate that leptin is a significant contributor to OA progression and serves as a potential mediator of the crosstalk between joint and brain.

## 3.4 Skeletal interoception

Interoception encompasses the complex processes of sensing, integrating, and interpreting signals emanating from the internal organs. Through interoception, the brain efficiently evaluates the physiological status of the viscera and releases regulatory signals accordingly. Interoception and exteroception form the adaptive behaviors to the internal systems and external environment [87]. In our prior article, we provided an exhaustive discourse on skeletal interoception and its integral involvement in the onset and progression of OA [19]. In brief, skeletal interoception is the process by which brain, after receiving and processing interoceptive signals from bone tissue, releases regulatory signals back to the original bone tissue, thereby participating in the modulation of bone homeostasis. The interoceptive circuit encompasses an ascending pathway, central processing, and a descending pathway. The ascending pathway is primarily facilitated by two routes: the dorsal root ganglia (DRG) of spinal nerves and vagal nerves. The descending pathways are constituted by the SNS and PSNS [15]. Notably, OBs produced PGE2 have been identified as the principal mediator of skeletal interoceptive signaling. The skeletal interoception is also called PGE2 interoception.

A study conducted in 2019 revealed that increased levels of PGE2 in osteoporotic bone could transmit signals to the hypothalamus via the prostaglandin E receptor 4 (EP4) on DRG sensory neurons. The subsequent upregulation of cAMP response element-binding protein (CREB) signaling in the VMH suppresses the activity of descending SNS. Considering the role of SNS in bone formation, this inhibition ultimately leads to an enhancement of bone formation metabolism [88]. NPY is also an important player in skeletal interoception. The interoceptive signals downregulate hypothalamic NPY levels, which promotes osteogenesis and lipolysis [89]. As bone is a mechanosensitive tissue that adapts to mechanical load variations through continuous remodeling, the reduction of PGE2 levels in response to unloading and its interoceptive effects suggest that mechanical stress is an additional trigger for PGE2-mediated skeletal interoception [90]. Given that heightened osteogenic activity in subchondral bone is a key pathological feature of advanced OA, and excessive mechanical stress is a significant etiological factor of OA, it is plausible that skeletal interoception contributes to the OA progression. Research indicates that PGE2 levels are elevated in the subchondral bone of the early destabilization of the medial meniscus (DMM) OA model, the inhibition of PGE2 levels and its EP4 receptors on sensory neurons markedly ameliorate OA symptoms and pathological alterations [91]. Recent studies by Gao et al. have shown that mechanical load-induced increase of PGE2 concentration in bone tissue promotes osteogenesis through the phosphorylation of CREB in the ARC of hypothalamus and the suppression of tyrosine hydroxylase (TH) expression in the PVN. Furthermore, elevated PGE2 levels are correlated with the progression of ankle OA and associated pain [20]. These findings preliminarily substantiate the role of skeletal interoception in bone metabolism and OA, underscoring the need for future research to elucidate the initiators of skeletal interoception and the critical brain regions involved in processing these signals.

**Figure 1.**
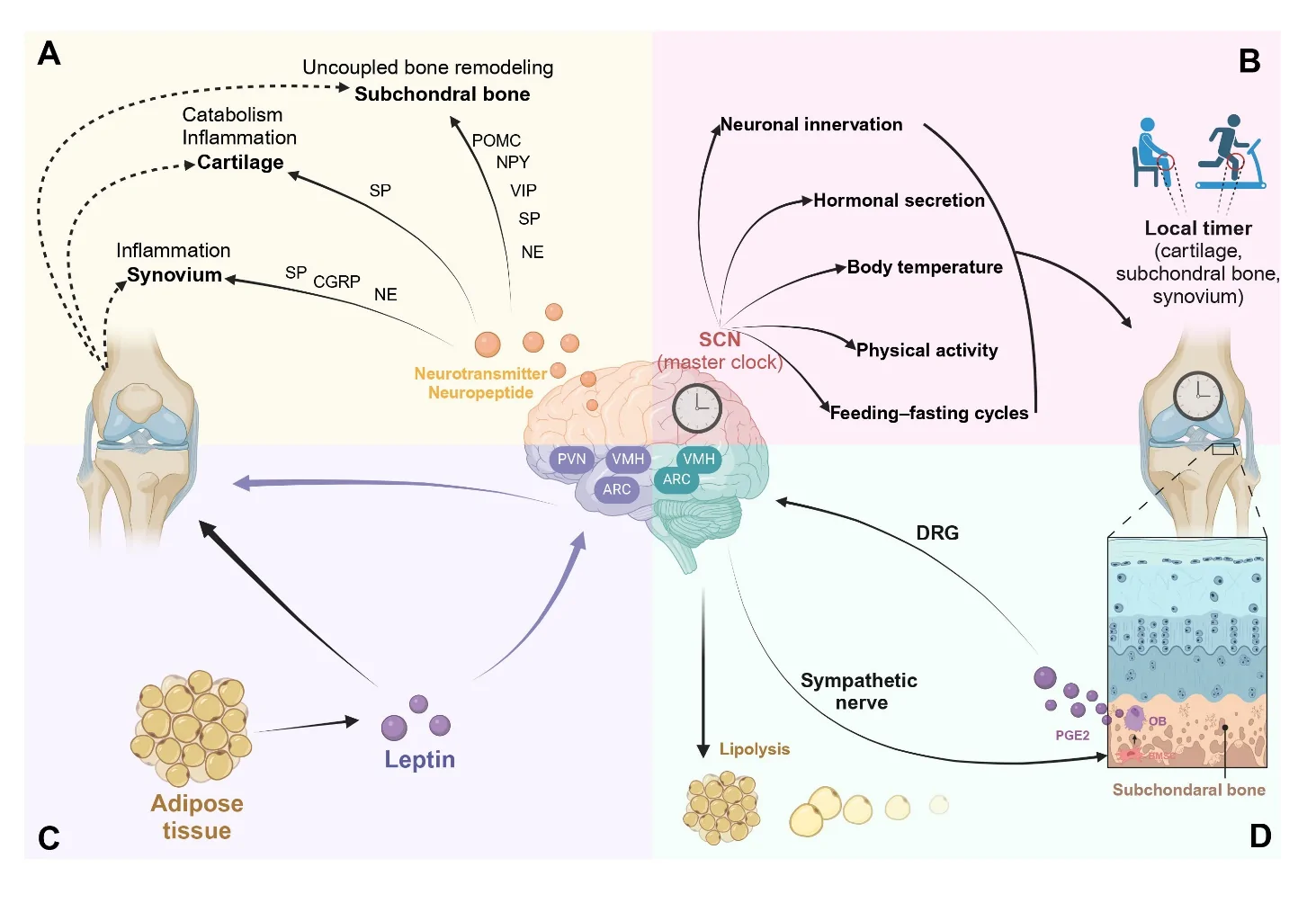
**The brain-joint crosstalk in OA progression**. (**A**) The neuroendocrine regulation of OA. Neurotransmitters and neuropeptides derived from the central and autonomic nervous systems exert effects on articular tissues, contributing to cartilage degeneration, synovial inflammation, and aberrant remodeling of the subchondral bone. (**B**) The circadian rhythm and OA. The SCN master clock impacts the local circadian rhythms of joint through the modulation of neural distribution, hormonal secretion, body temperature, feeding-fasting cycles, and physical activity. (**C**) Leptin: a potential mediator of crosstalk between joint and brain. Leptin, secreted by adipose tissue, can directly influence joint tissues and is implicated in the modulation of subchondral bone remodeling through its actions on the ARC, PVN, and VMH. (**D**) Skeletal interoception in OA. OBs within the subchondral bone, upon sensing mechanical load signals, produce PGE2. This molecule, upon interaction with the EP4 of the DRG, conveys interoceptive signals to the hypothalamic VMH and ARC, leading to a dampening of SNS activity. Consequently, this promotes the differentiation of BMSCs into OBs in subchondral bone and stimulates lipolysis to provide energy for osteogenesis. SCN, suprachiasmatic nucleus, ARC, hypothalamic arcuate nucleus, PVN, paraventricular nucleus, VMH, ventromedial hypothalamus, OB, osteoblast, PGE2, prostaglandin E2, EP4, prostaglandin E receptor 4, DRG, dorsal root ganglion, SNS, sympathetic nervous system, BMSC, bone marrow mesenchymal stem cell.

In summary, the CNS serving as the control center of the body, plays a pivotal role in the regulation of multiple organs and diseases. Its influence is manifested in several key domains: the regulatory effects of neurotransmitters and neuropeptides secreted by the CNS acting on their corresponding receptors, the governance of peripheral circadian rhythms by the cerebral circadian clock, the alterations in signaling following the action of leptin produced by adipose tissues on central receptors, and the burgeoning field of skeletal interoception that has gained attention in recent years (Fig. 1). Investigating the mechanisms through which the CNS exerts its regulatory effects on peripheral organs is instrumental in uncovering the patterns of disease and in the development of therapeutic strategies. More clinical studies are needed to delve into the therapeutic effects of targeting the CNS for OA, encompassing neuropeptides of the CNS and receptors. The role of modulating circadian rhythm and skeletal interoception in the treatment of OA should also garner attention. This will facilitate the translation of basic research findings into clinical applications.

## 4. The role of gut microbiota in OA pathogenesis

**Figure 2.**
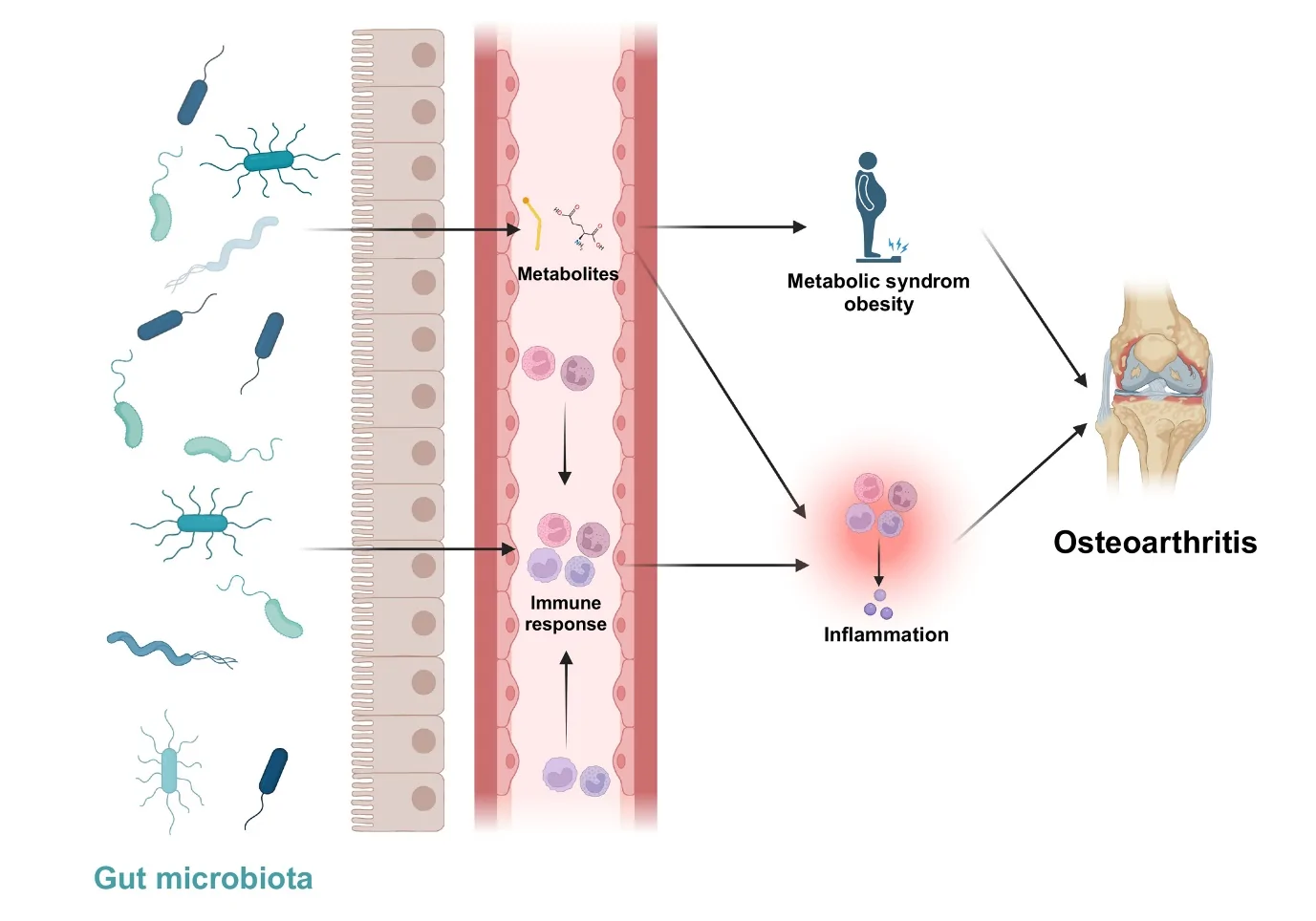
**The gut microbiota dysbiosis in the pathogenesis of OA**. Gut microbiota dysbiosis promotes the progression of OA through two primary mechanisms. Initially, the perturbation results in an elevated ratio of pro-inflammatory bacterial species, thereby triggering a systemic inflammation that fosters OA. Concurrently, metabolites derived from the gut microbiota facilitate the development of metabolic syndrome and obesity, which are established contributors to the pathogenesis of OA. Among these metabolites, there are also pro-inflammatory substances such as tryptophan and s SCFAs, which are instrumental in amplifying systemic inflammatory responses. SCFA, short-chain fatty acid.

The relationship between gut microbiota and disease has been a hot topic of research, focused on discerning the intricate causal mechanisms by which the gut microbiota may play a role in the etiology and treatment of diseases including OA. This research endeavors to explore the complex interplay between the intestinal microbial community and its potential influence on the onset and progression of pathological processes, thereby shedding light on novel perspectives for disease management and therapeutic development. The mechanisms by which the gut microbiota participate in joint destruction and pain remain complex and can be broadly categorized into two dimensions: firstly, the direct impact of systemic inflammation driven by gut microbiota dysbiosis on OA, and secondly, the indirect influence stemming from dysbiosis that fosters metabolic syndrome and obesity, consequently precipitating OA (Fig. 2). It is now recognized that systemic inflammation can be caused by gut microbiota dysbiosis, thereby leading to the progression of OA [92, 93]. Furthermore, the gut microbiota is implicated in metabolic pathways associated with amino acids, carbohydrates, and lipids, potentially facilitating the onset of OA [94, 95]. Collectively, the evidence points to a significant association between the gut microbiome and both the genesis and progression of OA, suggesting that modulation of the gut microbiota could present hopeful strategies for the prevention and management of OA. In this section, we will discuss the ecological changes in the gut microbiota of OA patients and animal models and the pathways through which they affect the emergence and progression of OA.

## 4.1 The overview of gut microbiome

The gut microbiome represents a sophisticated ecological system comprising all the coexisting unicellular life forms within the intestinal tract. This intricate assemblage of microorganisms encompasses about 10^13 cells, surpassing the collective count of the human body cells [96]. The genomic complement of these microbes is estimated to be approximately 100 times of the human genome; thus, they are also referred to as the "second genome of humans". This rich genetic reservoir plays a substantial role in modulating the comprehensive health profile of the host [97]. The constituents of the gut microbiota span a diverse array of more than 2000 species, encompassing bacteria, fungi, archaea, protists, and viruses. Predominantly, the bacterial community is characterized by four major phyla: Actinobacteria, Proteobacteria, Firmicutes, and Bacteroidetes [98], with the phyla Bacteroidetes and Firmicutes accounting for over 90% of the total bacterial count [99]. The gut microbiota is subject to modulation by a spectrum of factors, including pharmaceuticals, dietary habits, genetic predispositions, environmental exposures, chronological age, and the immune system [100-104]. Under normal conditions, the gut microbiota plays a crucial role in maintaining the host homeostasis: it ferments polysaccharides into absorbable monosaccharides, engages in the biosynthesis of essential vitamins such as B12, K, and B8, facilitates the generation and assimilation of fatty acids, modulates the host immune responses, and safeguards the integrity of the intestinal epithelium [105-107]. Conversely, dysbiosis of the gut microbiota has been implicated in a spectrum of diseases, ranging from cardiovascular conditions to cancer, autoimmune disorders, and psychiatric illnesses [103, 108-111].

## 4.2 Dysbiosis-induced systemic inflammation and OA

The term dysbiosis denotes perturbation in the composition and functionality of the gut microbiota. Dysbiosis is implicated in the pathogenesis of autoimmune and inflammatory conditions, including but not limited to rheumatoid arthritis, inflammatory bowel diseases, and ankylosing spondylitis [112, 113]. The potential mechanisms through which dysbiosis can instigate local or systemic inflammation are multifaceted and may encompass the following: the proliferation of pro-inflammatory microbes and the reduction of anti-inflammatory microbes, an escalation in the levels of pro-inflammatory metabolites such as tryptophan, short-chain fatty acids (SCFAs), and lipopolysaccharides, compromised intestinal barrier integrity [114, 115]. Elevated intestinal permeability facilitates the translocation of a greater number of bacteria and pro-inflammatory metabolites into the systemic circulation, which in turn augments the systemic inflammatory response [116, 117].

The prevailing consensus indicates that disruptions in the gut microbiota can incite immune responses and the ensuing systemic inflammation, thereby exacerbating OA [118, 119]. In a preclinical investigation conducted by Huang and colleagues, fecal microbiota from healthy subjects, individuals with knee OA without metabolic syndrome, and those with knee OA accompanied by metabolic syndrome were transplanted into germ-free mice two weeks prior to the induction of meniscal/ligamentous injury. The findings at the 8-week revealed that the microbiota from the cohort with knee OA and metabolic syndrome correlated with elevated levels of systemic inflammatory biomarkers, such as IL-1β and IL-6, alongside heightened intestinal permeability and exacerbated OA symptoms. Specifically, the prevalence of Ruminococcaceae and Faecalibacterium in the transplanted mice was positively associated with the severity of OA and the systemic inflammatory biomarker levels, whereas the abundance of Lachnospiraceae exhibited an inverse relationship. This research significantly elucidates that gut microbiota dysbiosis mediates OA via systemic inflammation and the implicated microbial components [120]. The fecal transplantation method used in this study can also be applied to the research of other diseases, providing a good paradigm for the study of the relationship between gut dysbiosis and diseases. Additional studies have echoed the correlation between systemic inflammation induced by dysbiosis and OA in murine models [121, 122]. However, the specific composition of the gut microbiota may also exert a protective effect on OA. Emmaline et al. utilized the Murphy Roths Large (MRL)/MpJ 'superhealer' and C57BL6/J (B6) wild-type mouse strains. By transplanting cecal material from MRL/MpJ mice into the DMM OA model of B6 mice, it was discovered that the MRL-into-B6 transplant significantly protected the mice from OA damage. The protective effects of microbiome transplantation in the DMM mouse model are linked to the increase in CD25+CD4+ T cells and a concurrent reduction in double-negative T cells [123]. This suggests that the OA-protective effect of MRL mice can be partially attributed to the gut microbiota, which can be transferred through microbiota transplantation, inducing systemic immune phenotype changes related to OA protection. The bidirectional role of the gut microbiota in OA largely depends on the composition of intestinal microbiome genera. Further exploration is needed in the future to identify microbiome species related to the progression or alleviation of OA, as well as to elucidate the molecular mechanisms mediating OA pathological changes.

Lipopolysaccharide (LPS), a constituent of the outer membrane of cell wall in Gram-negative bacteria, is known to trigger a systemic immune response. Studies have revealed a correlation between the levels of LPS in the bloodstream and synovial fluid with the extent of knee joint destruction and the severity of knee pain in OA patients [119, 124, 125]. This correlation underscores the link between gut microbiota dysbiosis and OA at a population level, which is supported by the refined experimental design and appropriate statistical methods. In conclusion, an array of studies has demonstrated a notable association between gut microbiota dysbiosis and the presence of OA. However, the investigation into the mechanisms underlying this association is still in its nascent stages.

## 4.3 Dysbiosis-induced metabolic disorders and OA

OA is a metabolic-related disease, manifesting in both weight-bearing and non-weight-bearing joints. The onset of OA is associated with various metabolic disorders, such as obesity and type 2 diabetes [126]. A cross-sectional study based on populations from South Korea, North America, and Canada have all indicated a correlation between metabolic disorders and the risk of OA. Moreover, these metabolic disruptions are implicated in hindering functional recovery after joint replacement surgery [127, 128].

Obesity is an important risk factor for the advancement of OA [129], as it not only amplifies the mechanical stress on weight-bearing joints, but also involves adipose tissue-derived molecules such as leptin in joint destruction processes. Current research posits that an obesity model induced by a high-fat diet leads to alterations in the gut microbiota composition of mice, with a notable increase in Bifidobacteria and several pro-inflammatory bacterial genera [121]. Consequently, metabolic disorders precipitated by gut microbiota dysbiosis are substantial contributors to the genesis and exacerbation of OA. A preclinical study by Huang et al. has fully substantiated this conclusion. Metabolic syndrome is a cluster of closely related clinical diseases, including impaired glucose tolerance, central obesity, insulin resistance (IR), hypertension, and dyslipidemia [130]. By transplanting feces from healthy individuals and those with metabolic syndrome into DMM mouse models, the study revealed that the fecal microbiota from metabolic syndrome patients correlated with increased severity of OA and heightened levels of intra-articular and systemic inflammatory biomarkers, including IL-1β, IL-6, and macrophage inflammatory protein-1α (MIP-1α) [120]. Guss et al. further elucidated the significant correlation between metabolic syndrome induced by alterations in the gut microbiota and OA. They utilized Toll-like receptor-5 deficient (TLR5KO) mice, which exhibit altered gut microbiota, to induce metabolic syndrome, and administered chronic antibiotics to TLR5KO mice to prevent metabolic syndrome (TLR5KOΔMicrobiota). Additionally, cyclic compressive loading was applied to induce OA. It was observed that at 6 weeks, compared to the normal control group, TLR5KO mice exhibited more pronounced cartilage damage, while TLR5KOΔMicrobiota mice showed weaker cartilage damage than other groups. The composition of the gut microbiota in each group of mice was distinct, revealing the potential impact of microbiota dysbiosis-induced metabolic syndrome on cartilage pathology [122]. Beyond obesity, gut microbiota dysbiosis is also involved in the development of diabetes [131], suggesting that targeted modulation of the gut microbiota could offer therapeutic benefits not only for metabolic syndrome but also for OA associated with metabolic syndrome. In a cohort study involving 1,359 participants, it was found that those with symptomatic hand osteoarthritis (SHOA) exhibited significant alterations in microbial functions related to tryptophan metabolism. Moreover, lower levels of indole-3-lactic acid (ILA) in plasma were associated with SHOA. This study reveals a potential link between tryptophan metabolism associated with gut microbiota and OA [132]. However, to avoid potential selection bias, the authors did not adjust for other metabolites/microbiomes. Consequently, some of the observed associations in the results may be confounded by other plasma metabolites that occur prior to the assessed risk factors and are also related to SHOA. Further research is needed to confirm and explore this association.

While a substantial body of research has confirmed the potential link between gut microbiota dysbiosis and OA, some studies have yielded contradictory conclusions. In the latest study by Christina et al., they categorized 93 pet dogs into a healthy group and an OA pain group and collected their fecal samples to observe differences in fecal microbial communities. The results revealed no significant differences in alpha and beta diversity between the two groups. No differences in the Firmicutes to Bacteroidetes ratio or alpha or beta diversities were found regarding pain severity or activity level [133]. This study discusses the association between OA pain and the composition of the gut microbiota in naturally occurring OA, whereas previous research has primarily focused on exploring the relationship between gut microbiota dysbiosis and pathological changes in OA, especially metabolism-related OA. Even though Boer et al. previously reported a significant association between the abundance of fecal Streptococcus species and OA-related knee pain [134], such findings are not always reproducible. Although specific gut microbiota characteristics may not consistently differentiate between individuals with and without OA, subtle shifts in the microbiome could alter the translocation of gut microbes or microbial products. Elevated levels of LPS and LPS-binding protein (LBP) are two biomarkers often used to indicate a compromised intestinal barrier function, which are associated with an increase in the severity of human OA [120]. In this study, there are several limitations that might affect the research outcomes: dogs with OA pain are significantly older than those without OA pain, dogs were not stratified by breed within each group, making breed a potential confounding variable, and the sample size is relatively small, with an imbalance between the healthy group and the OA pain group. Therefore, even in the face of numerous studies confirming that gut microbiota dysbiosis is one of the risk factors for OA progress, further research is needed to explore the association between the composition of the gut microbiota and OA pain.

## 5. Sarcopenia: a potential risk factor for OA

**Figure 3.**
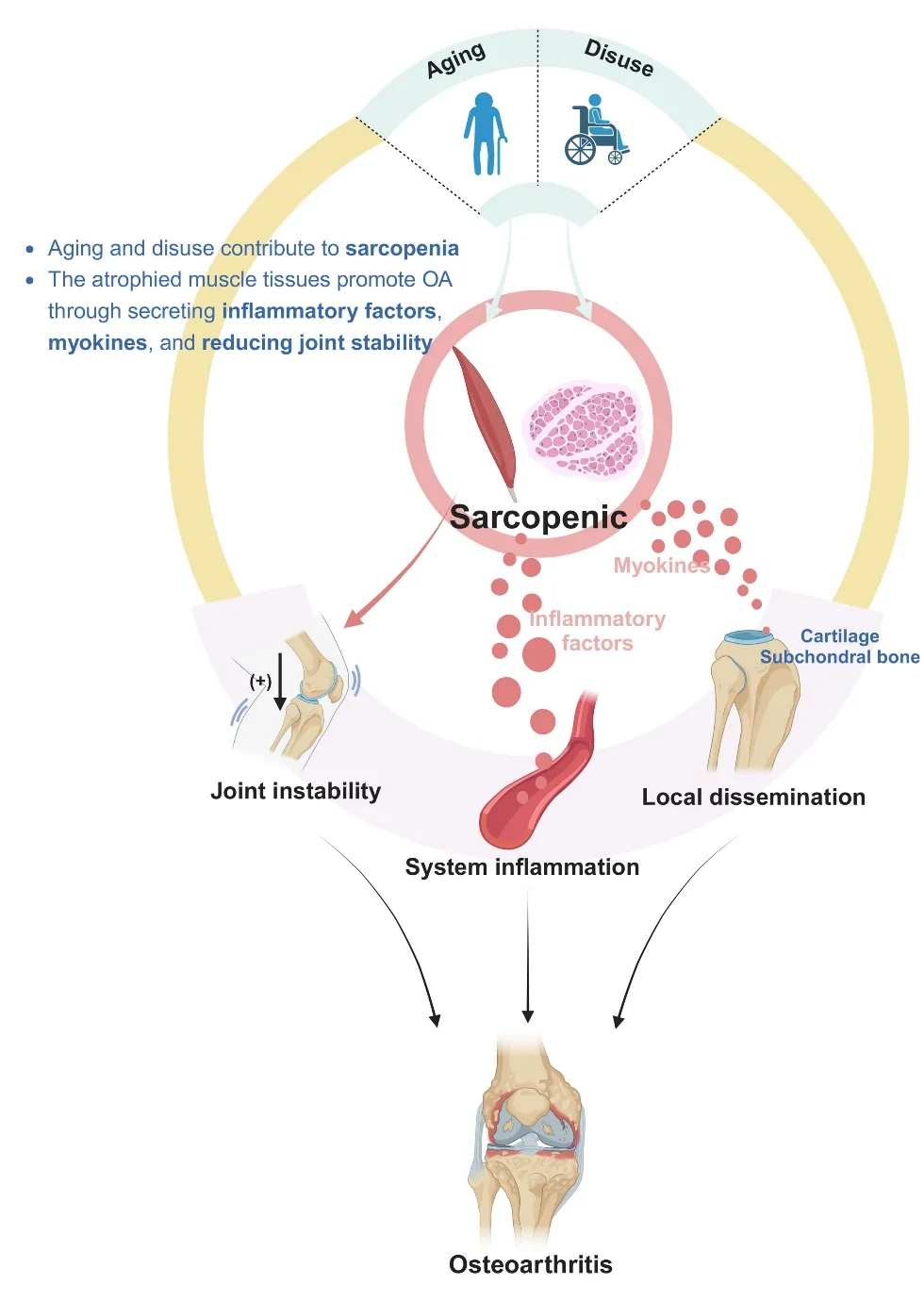
**The potential role of sarcopenia in the etiology of OA**. The risk of sarcopenia rises with aging and muscle disuse. Decreased strength in the periarticular muscles can lead to joint instability, subjecting the joints to increased mechanical stress. Furthermore, the secretion of inflammatory cytokines by atrophied muscle tissue enters systemic circulation, potentially propagating systemic or local inflammation. Myokines released from the muscle may also exert paracrine effects, diffusing to the adjacent cartilage or subchondral bone and influencing their metabolic homeostatic and remodeling processes. Above mechanisms may increase the risk of OA associated with sarcopenia.

Sarcopenia was initially characterized by Irwin Rosenberg in the 1980s to describe age-related muscle loss [135]. Sarcopenia is currently recognized as a syndrome encompassing the progressive diminution of muscle mass, strength, and functionality with aging [136]. This condition impacts roughly 10%-16% of elderly individuals globally [137]. OA and sarcopenia are both aging-related disorders. They have been increasingly linked, with recent research indicating that sarcopenia is a notable risk factor for the onset of OA [138-141]. Although some of these studies are based on mendelian randomization methods or retrospective questionnaire data, which may introduce potential recall or information biases and residual confounding, they provide a foundation for future research directions. Jelle and colleagues, through differential and enrichment analysis of the transcriptome of the vastus lateralis muscle from older and younger humans, discovered significant enrichment of OA pathways [142]. The mechanisms through which sarcopenia may exacerbate OA are multifaceted, involving weakened mechanical support, metabolic dysregulation, and heightened inflammatory activity (Fig. 3). In this section, we will explore the existing research that delves into the interplay between sarcopenia and OA, with a particular emphasis on the potential mechanisms by which sarcopenia could foster the development and progression of OA. We will specifically address how sarcopenia influences bone metabolism and modulates inflammatory responses, which are critical in the pathogenesis of OA.

## 5.1 Joint instability

In patients with sarcopenia, the diminished supportive effect of muscles on joints and subsequent abnormal joint loading are the most recognized mechanisms by which sarcopenia may precipitate OA. Weight-bearing joints are essentially cavitary structures encased by surrounding muscular support, and the strength of surrounding muscles affects the load on the joint. Abnormal or excessive joint loading can exacerbate cartilage shear stress and attrition [143, 144]. Moreover, such excessive loads can induce pathological bone remodeling in subchondral bone [12, 20], leading to pathological changes such as osteophyte formation and thickening of the subchondral bone plate in the later stage of OA [145]. In individuals with sarcopenia, the increased instability of the knee joint results in the transmission of aberrant mechanical loads to the articular cartilage and subchondral bone. To counteract this, clinicians frequently advocate for muscle strengthening exercises to augment joint stability and mitigate the progression of OA. Research has demonstrated that rehabilitative training focusing on the knee joint can markedly enhance lower limb muscle strength in OA patients [146]. Additionally, studies have indicated that isokinetic muscle strengthening, and neuromuscular exercises can improve joint stability, leading to a significant reduction in pain and functional limitations associated with OA [147, 148]. Nonetheless, there is a recognized need for further biomechanical research to provide a more objective assessment of the influence of sarcopenia on joint loading and stability, thereby offering a clearer understanding of its role in the pathophysiology of OA.

## 5.2 Sarcopenia induced system inflammation

OA is recognized as a chronic inflammatory condition that encompasses both local articular and systemic manifestations [149, 150]. Sarcopenia is accompanied by systemic inflammation, which is an integral part of its pathophysiological process. Research has identified correlations between serum levels of cytokines such as IL-16, IL-12, and IL-5 with grip strength [151], and has reported elevated levels of inflammatory markers in the peripheral blood of sarcopenic patients, including TNF-α, IL-1β, IL-4, IL-6, and IL-10 [152, 153]. Our recent unpublished research, entailing a differential gene expression and enrich analysis of the gastrocnemius muscle RNA sequencing in rats at 6 and 24 months (GEO dataset: GSE118825), has uncovered the enrichment of various inflammatory pathways such as granulocyte migration, leukocyte chemotaxis, and granulocyte chemotaxis. Although the precise mechanisms linking sarcopenia to systemic inflammation remain to be fully elucidated, it is hypothesized that mitochondrial dysfunction in aging muscle fibers, reduced β-oxidation capacity, and heightened reactive oxygen species (ROS) production may contribute to a state of chronic systemic inflammation [154-156]. Conversely, the exacerbation of systemic inflammation with aging is also considered a significant factor in the etiology of sarcopenia, indicating a potential reciprocal aggravation between the two conditions [157, 158].

Elucidating the interplay between sarcopenia and systemic inflammation is crucial for not only identifying novel inflammation-related targets for the treatment of sarcopenia but also understanding the relationship between sarcopenia and OA. It is plausible that peripheral inflammatory mediators associated with sarcopenia could access joint tissues via the bloodstream and contribute to the maintenance of chronic inflammatory states within OA joints. Subsequent in vivo and in vitro experimental designs should aim to validate the role of sarcopenia associated inflammation in OA. Such studies could offer substantial insights into OA etiology and pave the way for innovative therapeutic approaches.

## 5.3 The potential role of myokines in OA progression

In the past two decades, the endocrine role of skeletal muscle has been increasingly acknowledged. The skeletal muscle can also be considered an endocrine organ [159]. Pedersen were the first to identify the term "myokine", stemming from their discovery that muscles release IL-6 upon contraction [160]. Muscle tissue has been shown to secrete an array of hormones and cytokines that contribute to various physiological processes, including metabolism, immune response, tissue repair, and even vascular remodeling [161-163]. As adjacent tissues to skeletal muscle, joints may be subject to the endocrine effects of muscle tissue through two primary mechanisms. Firstly, the secretions of muscle tissue can potentially modulate cartilage metabolism in the vicinity. Dana and colleagues demonstrated that co-culture of C2C12 muscle cells with chondrocytes enhanced the synthesis of collagen II and IX proteins by chondrocytes [164]. This finding implies that in sarcopenia, the senescence and diminished numbers of muscle cells could adversely impact the anabolism of cartilage through these paracrine effects.

Secondly, bones and muscles are closely interconnected. The bone-muscle crosstalk has been preliminarily explored. We speculate that muscle tissues may participate in the progression of OA by affecting the metabolism of subchondral bone. Emerging research indicates that the crosstalk between muscle and bone occurs via multiple pathways, which are essential for the preservation of bone mass. Myokines such as fibroblast growth factor 21 (FGF21), IL-6, myostatin, IL-10, and irisin are released from muscles and affect bone metabolism [165]. Recent findings have unveiled a novel axis of communication between muscle and bone through the folliculin interacting protein 1 (FNIP1)- transcription factor EB (TFEB)- insulin like growth factor 2 (IGF2) pathway, which is implicated in bone metabolic processes [166]. Additionally, the extracellular vesicles originating from skeletal muscle (Mu-EVs) have been shown to transport glycolytic enzymes that facilitate bone anabolism. Further exploration has revealed that Mu-EVs promote glycolysis in BMSCs by delivering lactate dehydrogenase A, thereby stimulating bone formation [6]. The potential for atrophied muscles to exacerbate OA progression is heightened by the concomitant osteoporotic changes in the surrounding bone, given that osteoporosis is regarded by some researchers as a latent risk factor for OA [167-170]. Many studies have demonstrated the efficacy of osteoporosis therapeutics in the treatment of OA as well [171]. The interaction between muscle and bone is complex, it is worth further exploring whether muscles also participate in the regulation of bone catabolism. Elucidating the influence of muscle-derived myokines on subchondral bone metabolism and their contribution to OA development is pivotal for a comprehensive understanding of OA pathogenesis and the interrelationship between sarcopenia and OA. This knowledge may unlock novel preventive and therapeutic strategies that target the muscle-bone axis in OA and sarcopenia.

In the end, although the majority of studies posit that sarcopenia is a potential contributing factor to OA, there is research that refutes this correlation. A multicenter observational study by Devyani et al. found that sarcopenic obesity, rather than sarcopenia alone, is a risk factor for OA [172]. However, the subjects of this study were primarily Caucasian, the results may not be generalizable to other racial populations. As with any observational study, there is a possibility of residual confounding. These limitations may account for the inconsistencies between this study and others. In summary, research on the association between sarcopenia and OA is still in its infancy, further studies are needed to confirm this relationship to explore the underlying mechanisms. For example, by examining OA phenotypes within animal models of sarcopenia and by assessing the levels of inflammatory and myokine factors both locally and in circulation, it is possible to establish potential correlations between sarcopenia and OA. This approach can provide valuable insights into the underlying mechanisms connecting these two conditions.

## 6. Targeting organ-joint axes is a promising therapy for OA and its pain

The common clinical treatments for early-stage OA patients include oral administration of glucosamine, nonsteroidal anti-inflammatory drugs (NSAIDs), intra-articular injection of hyaluronic acid, functional exercises, and physical therapy. For severe, end-stage OA patients, joint replacement surgery is typically recommended to alleviate pain and improve quality of life. The investigation into the etiology and treatment of OA has been ongoing for many years, yet the complexity of its pathogenesis makes it challenging for single-modality therapies to achieve optimal treatment efficacy. Previous research has predominantly concentrated on elucidating the molecular biological mechanisms within the affected tissues of OA, aiming to target specific molecules in cartilage, subchondral bone, or synovium to achieve therapeutic objectives. However, the vast number of substances and molecules represents one of the most significant challenges for researchers. Numerous studies have introduced innovative biomaterials integrated with bioactive molecules, which may offer potential benefits for articular cartilage repair and the management of synovial inflammation. Nevertheless, these studies have often disregarded the intricate interplay between joint tissues and other organs within the body. As previously highlighted, biological alterations in other tissues or organs, such as the brain, gut, and muscles, could influence distant joint tissues through specific mediators. Targeting the inherent biological processes of these organs or their mediators could potentially impact the pathological changes in OA, leading to therapeutic effects. In pursuit of more effective OA treatments, we could view humans as a unified system and explore the pathogenesis and treatment of OA from a broader, more holistic perspective, which may lead to unforeseen therapeutic breakthroughs. To date, by targeting circadian rhythms, skeletal interoception, and the gut microbiota, some preclinical studies and a limited number of clinical studies have shown initial success. This offers hope for more effective treatments for OA in the future.

## 6.1 Regulate central and local circadian rhythms

Circadian genes within articular tissues are subject to the control of central master clock and are also modulated by local timers. With aging, the regulatory efficacy of these genes may decline. This reduction is linked not solely to the physiological transformations inherent to the aging process but also to an enhanced vulnerability to OA [173]. Consequently, targeted interventions in the systemic or localized circadian system could potentially open novel avenues for therapeutic intervention. Such approaches may ameliorate the impacts of senescence and diminish the risk factors associated with OA, offering promising strategies for the treatment and prevention of age-related joint degeneration.

Recent scientific discoveries have further underscored the pivotal role of circadian rhythms in maintaining tissue homeostasis and adapting to environmental changes. Within joint tissues, the expression profiles of circadian genes are markedly affected by daily activity patterns, potentially exerting profound implications for the adaptability and self-repair capacity of joints. Consequently, the modulation of circadian rhythms offers a novel perspective for both the prevention and therapeutic intervention of OA. The circadian patterns of mechanical stress and the concomitant daily oscillations in osmotic pressure offer precise temporal cues essential for synchronizing the diurnal rhythms of cartilage [76]. Engaging in regular and moderate physical activity as part of a daily routine may facilitate the resynchronization of the joint circadian rhythms, thereby exerting a beneficial influence on the joint health [174, 175]. Moreover, adopting other consistent lifestyle practices such as a regular dietary regimen, may enhance systemic temporal cues, which could ameliorate or even reverse the deterioration of circadian rhythm function that occurs with advancing age. Pharmacological intervention represents a burgeoning therapeutic strategy that is under intensive investigation. Certain small molecule entities have been discovered to regulate the activity of genes associated with circadian rhythms, presenting innovative avenues for the development of treatments that target the biological clock [176-178]. For example, under the treatment of nuclear receptor REV-ERBα/β agonists, alterations in the expression of multiple core clock genes in mice have been observed, including *Per2*, *Crytochrome 2* (*Cry2*), *Bmal1*, *Neuronal PAS domain-containing protein 2* (*Npas2*), and *Clock* [179]. While the enduring efficacy and safety profiles of these interventions await validation through clinical trials, they hold promise for the advancement of personalized OA therapies. Future studies should delve deeper into the intricate interplay between circadian rhythms and joint health, encompassing the expression patterns of clock genes within articular tissues, the impact mechanisms of mechanical stimuli on the biological clock, and the molecular mechanisms of pharmacological interventions. Adopting an integrative, multidisciplinary approach to research such as bioinformatics, genetics, and exercise physiology, will be pivotal in enhancing our grasp of the complexities inherent in this domain. Such a strategy will not only broaden the scope of investigation but also enrich the depth of our insights, allowing for a more nuanced appreciation of the interplay between biological rhythms, genetic factors, and physical activity as they pertain to joint health and the pathogenesis of conditions like OA. By leveraging the power of big data analytics, genetic sequencing, and physiological assessments, researchers can uncover novel biomarkers, identify genetic predispositions, and optimize exercise interventions to promote more effective and personalized treatment strategies.

## 6.2 Target skeletal interoception

As previously discussed, skeletal interoceptive signals are mediated by PGE2. Hence the term skeletal interoception is also called PGE2 interoception [15]. In our prior publication, we delineated the synthesis, secretion, and physiological roles of PGE2, and comprehensively discussed the role of PGE2 in the metabolism of OA cartilage, subchondral bone, and synovium [22]. Targeting PGE2 has been shown to inhibit the progression of OA and associated pain through a multitude of mechanisms, such as targeting skeletal interoception to inhibiting abnormal subchondral bone remodeling. Current studies have established that PGE2 concentrations are markedly elevated in the subchondral of both spontaneous OA mouse models and those induced by DMM. The administration of celecoxib, a COX-2 inhibitor, has been demonstrated to significantly ameliorate disease progression in these mouse models [91, 180]. Correspondingly, the selective deletion of the EP4 on sensory neurons has been shown to exert analogous effects in pre-clinical models [91]. Elevated levels of PGE2 have also been correlated with ankle OA and pain. The CNS can perceive the increase in PGE2 levels in the subchondral bone due to mechanical loading. In response, brain dispatches regulatory signals that increase osteoblastic activity, thus contributing to the progression of ankle OA [20]. This mechanism leads us to surmise that skeletal interoception potentially participates in the onset and progression of OA across all weight-bearing joints, such as knee, hip, and ankle. Celecoxib may have a significant therapeutic impact in the mitigation of symptoms and pain related to weight-bearing OA, offering a crucial intervention for these debilitating conditions.

Targeting PGE2 interoception presents a promising therapeutic strategy for OA. The interoceptive role of PGE2 in subchondral bone and bone metabolism is pivotal, as it influences the progression of OA and associated pain. Recent research has indicated that modulating PGE2 levels through using celecoxib can lead to a sustained analgesic effect and potentially mitigate the progression of intervertebral disc degeneration (IDD). The innovative approach of using a low dose (20 mg·kg-1 per day) of celecoxib has been shown to maintain physiological concentrations of PGE2 in the endplates, which is crucial for bone health. This treatment not only reduces spinal pain but also prevents the recurrence of pain after treatment cessation [181]. The mechanism behind this involves the reduction of endplate porosity, which in turn limits excessive sensory nerve innervation—a key factor in OA pain. The pathogenesis and pathological changes of IDD share similarities with those of OA. By maintaining the homeostasis of PGE2, low-dose celecoxib treatment may inhibit the progression of OA and provide long-lasting pain relief.

The interaction between PGE2, OBs, OCs, and sensory nerves is complex and critical in the pathophysiology of OA. The activation of OCs by PGE2 leads to bone resorption and contributes to the innervation of sensory nerves in subchondral bone [182]. Targeting this pathway, for instance, by inhibiting the activity of OCs or production of netrin1, a factor that promotes nerve sprouting, could offer additional therapeutic benefits [183, 184]. The multi-targeted approach of modulating PGE2 interoception not only addresses pain relief but also impacts bone remodeling, offering a comprehensive strategy for managing OA. Overall, targeting PGE2 and sensory nerves related to skeletal interoception presents substantial potential for the therapeutic intervention of OA. Nonetheless, existing research is predominantly limited to the preclinical stage. It is imperative for future studies to initiate extensive clinical trials aimed at assessing the therapeutic efficacy of various dosages of celecoxib on OA, with a focus that transcends mere analgesic effects and delves into the modulation of disease progression. Such trials would provide invaluable insights into the role of PGE2 inhibition in the comprehensive management of OA, offering a more nuanced understanding of its potential as a disease-modifying strategy.

## 6.3 Regulate gut microbes

Targeting the gut microbiome in the treatment of OA is an innovative strategy that hinges on the growing evidence suggesting a "gut-joint axis". This approach recognizes the potential of gut microbiome to modulate systemic inflammation, which is a key factor in OA pathogenesis. Therapies that aim to restore a balanced gut microbiota could alleviate OA symptoms and potentially modify disease progression [185].

One avenue is the use of probiotics, when administered in adequate amounts, confer a health benefit on the host [186]. Certain strains of probiotics, such as *Lactobacillus, Bifidobacterium* and *Streptoccocus thermophilus*, have demonstrated the ability to alleviate joint pain and OA progression in clinical trials [187-189]. These beneficial bacteria may exert their effects by producing anti-inflammatory metabolites, enhancing the intestinal barrier function, and regulating immune responses. The beneficial microbes can suppress inflammatory pathways, such as the cyclic GMP-AMP synthase (cGAS)-stimulator of interferon genes (STING) pathway, and inhibit the infiltration of inflammatory cells, including pro-inflammatory macrophages and CD8+ T cells, which contributes to the maintenance of local immune homeostasis [190-192]. Another therapeutic strategy is the employment of prebiotics, which are non-digestible food components that selectively stimulate the growth and activity of beneficial gut bacteria [193]. Prebiotic fibers, such as inulin and oligofructose, can increase the production of SCFAs, which have anti-inflammatory properties and improve metabolic health [194, 195]. Dietary interventions are also critical, as specific nutrients can influence the composition of the gut microbiome. High-fiber diets, rich in fruits, vegetables, and whole grains, can promote the growth of beneficial bacteria and increase SCFA levels, thereby potentially reducing OA-associated inflammation and pain [196]. Moreover, the modulation of gut microbiome through physical activity is an emerging area of interest. Exercise enhances gut microbiome diversity and function, potentially leading to the improvements in joint health [197].

Traditional Chinese medicine (TCM) encompasses a variety of prescriptions and therapeutic strategies that hold potential therapeutic benefits in OA [198-200]. Some of these TCM approaches alleviate OA symptoms by modulating the gut microbiota, such as Guizhi Shaoyao Zhimu Decoction (GSZD) and moxibustion. Traditional Chinese medicinal formulas are complex in composition and rich in functions, capable of acting on multiple organs and targets. For instance, Meng et al. have characterized 19 chemical constituents within GSZD, which significantly attenuate the abundance of pro-inflammatory microbes in the gut microbiome of arthritis rats and reduce the serum levels of key inflammatory mediators, including lipopolysaccharide, TNF-α, and interleukin [201]. Therapeutic moxibustion has been reported to ameliorate the pathological manifestations in an OA murine model, encompassing subchondral bone sclerosis, cartilage degradation, and synovial inflammation. Additionally, moxibustion treatment has been shown to decrease the abundance of Rumin-ococcaceae and Proteobacteria in the gut, aligning the microbiota profile more closely with that of control mice [202]. These findings suggest that TCM exerted beneficial effects on diseases such as OA by modulating the structure and composition of gut microbiota. The action modalities within TCM are complex, necessitating additional research to delve deeper into the vast array of therapies and their mechanisms of action. Such exploration is vital for advancing our understanding of TCM efficacy and for integrating these ancient healing practices with modern medical approaches. By elucidating the active components and their specific targets, we can enhance the potential of TCM contributing to evidence-based healthcare solutions.

Despite these promising therapeutic avenues, challenges exist including the need for a deeper understanding of the complex interactions between the gut microbiome and OA. Personalized medicine approaches that consider individual variations in gut microbiome composition and function may be necessary to optimize treatment efficacy. All in all, targeting the gut microbiome in OA treatment is a burgeoning field with the potential to offer new avenues for managing this debilitating disease. However, further research is required to fully elucidate the mechanisms at play, and to develop targeted and effective therapies.

## 7. Conclusion and prospects

In conclusion, this comprehensive review discussed the complex organ-joint axes in the etiology and pathogenesis of OA, transcending the traditional focus on localized joint pathology. The complex interplay among the brain, gut microbiota, muscle, and joint tissues presents a paradigmatic shift towards a more holistic comprehension of OA. This systemic perspective not only illuminates the multifactorial origins of OA but also paves the way for innovative therapeutic strategies that target organ crosstalk. The role of the brain in regulating joint health through neuroendocrine pathways is particularly noteworthy. The discovery of the brain's influence on subchondral bone remodeling and pain perception via neuromediators like SP and CGRP opens up new avenues for OA pathogenesis. Furthermore, the impact of circadian rhythm on joint homeostasis suggests that chronotherapy could be a potent approach to synchronize treatment with the body's natural rhythms, potentially enhancing the efficacy of OA management. Preclinical experiments have shown that targeting skeletal interoception can markedly suppress the advancement of OA and offer enduring alleviation of pain following the discontinuation of treatment. This surprising discovery has shed new light on the pharmacological profile and mechanisms of celecoxib. It suggests that celecoxib may exert its therapeutic benefits not only the analgesia effects but also through modulation of skeletal interoception, potentially offering a novel dimension to its use in the management of OA.

The emerging role of gut microbiome as a modulator of systemic inflammation and metabolic disorders related to OA cannot be overstated. The "gut-joint axis" hypothesis is supported by evidence showing that dysbiosis can precipitate system inflammation and metabolic imbalances, both of which are implicated in OA progression. Targeting the gut microbiome with probiotics, prebiotics, and dietary modifications represents a promising preventive and therapeutic strategy that could mitigate OA symptoms by rectifying the underlying systemic inflammation. The identified sarcopenia link to OA pathogenesis underscores the importance of muscle health in joint integrity. Interventions aimed at preserving muscle mass and strength, such as resistance training and nutritional support, may not only delay the onset of sarcopenia but also reduce the risk of OA. The exploration of myokines and their crosstalk with bone metabolism presents a novel frontier in the development of therapies for muscle and joint health.

Looking forward, the prospects for OA treatment are both challenging and exciting. A deeper dissection of the molecular and cellular mechanisms underpinning the organ crosstalk is essential for devising targeted therapies. The advent of precision medicine, with its emphasis on individual variability, offers a tailored approach to treating OA by considering personal genetic, metabolic, and microbiome profiles. Clinical trials incorporating multi-targeted therapies that address the brain-joint, gut-joint, and muscle-joint axes are warranted. These trials should evaluate the long-term benefits and safety of interventions such as low-dose celecoxib, personalized probiotic treatments, and lifestyle modifications that enhance gut microbiome diversity and function. Moreover, it is essential to delve deeper into the role of environmental factors, such as diet and physical activity in shaping the gut microbiome, and to assess their potential impact on the management strategies for OA. The integration of TCM and modern therapeutics could provide a holistic approach to OA treatment, harnessing the synergistic effects of various treatment modalities. It should be emphasized that the mechanisms of organ-joint interaction are complex. An interdisciplinary approach is required to study OA, incorporating fields such as neurology, endocrinology, microbiology, biomechanics, and orthopedics. Enhancing interdisciplinary collaboration is essential to achieve broad insights, comprehensive research methodologies, and robust research results.

## Data Availability

No data was used for the research described in the article.
